# An unusual case of a microscopic alveolar adenoma coexisting with lung carcinoma: a case report and review of the literature

**DOI:** 10.1186/1752-1947-5-187

**Published:** 2011-05-18

**Authors:** Tapan Bhavsar, Guldeep Uppal, John M Travaline, Colleen Gaughan, Yajue Huang, Jasvir S Khurana

**Affiliations:** 1 Department of Pathology and Laboratory Medicine, Temple University Hospital, Philadelphia, PA, USA; 2 Department of Medicine, Temple University Hospital, Philadelphia, PA, USA; 3 Department of Cardiothoracic Surgery, School of Medicine, Temple University, Philadelphia, PA, USA

## Abstract

**Introduction:**

Alveolar adenomas are extremely rare, benign, primary lung tumors of unknown histogenesis that are characterized by proliferative type II alveolar epithelium and septal mesenchyma. Mostly incidental, they are clinically important as they can imitate benign primary and secondary malignant tumors and at times are difficult to differentiate from early-stage lung cancer. We describe the case of a 59-year-old man with an incidental microscopic alveolar adenoma coexisting with poorly differentiated lung carcinoma.

**Case presentation:**

A 59-year-old Caucasian man with a medical history of smoking and chronic obstructive pulmonary disease was incidentally found to have a right upper lobe mass while undergoing a computed tomographic chest scan as part of a chronic obstructive pulmonary disease clinical trial. Our patient underwent a right upper lobectomy after a bronchoscopic biopsy of the mass revealed the mass to be a carcinoma. A pathological examination revealed an incidental, small, 0.2 cm, well circumscribed lesion on the staple line margin of the lobectomy in addition to the carcinoma. Histopathological and immunohistochemical examinations revealed the lesion to be an alveolar adenoma.

**Conclusions:**

We report the rare presentation of a microscopic alveolar adenoma coexisting with lung carcinoma. Alveolar adenoma is an entirely benign incidental neoplasm that can be precisely diagnosed using immunohistochemical analysis in addition to its unique histopathological characteristics.

## Introduction

Alveolar adenomas (AAs) or 'Yousem's tumors', first described by Yousem and Hochholzer in 1986 [1], are pulmonary tumors of uncertain histogenesis. Several types of pulmonary adenomas are recognized by the revised World Health Organization/International Association for the Study of Lung Cancer classification [2]. AAs are characterized by proliferation of type II alveolar epithelium and septal mesenchyma [3]. They are histologically benign, and recurrence after resection has never been reported [4-6]. AAs are detected incidentally as isolated coin lesions on routine chest radiographs [4,7]; however, at times, they are difficult to differentiate from the early stages of lung cancer. They usually occur in the middle and lower lobes of middle-aged and older women [8]. AAs have a characteristic multi-cystic histology resembling normal lung parenchyma. They are well demarcated peripheral pulmonary lesions composed of a network of spaces lined with simple cuboidal epithelium that contain stroma ranging from thin, inconspicuous strands of connective tissue to broad accumulations of spindle cells accompanied by a myxoid matrix [4]. A review of the English language medical literature revealed that fewer than 30 cases of AA have been reported to date [1,4-6]. We describe the rare case of an incidental, microscopic (0.2 cm) AA coexisting with lung carcinoma.

## Case presentation

A 59-year-old Caucasian man with a medical history of smoking and chronic obstructive pulmonary disease (COPD) who was incidentally found to have a right upper lobe mass while undergoing computed tomography (CT) of the chest as part of a clinical trial for COPD. A positron emission tomographic scan demonstrated fluorodeoxyglucose avidity of a suprahilar right upper lobe mass without any mediastinal lymphadenopathy. Our patient underwent a bronchoscopic biopsy of the mass, which revealed it to be a carcinoma, and he was referred for surgery. Our patient then underwent a right upper lobectomy and tolerated the procedure well without any remarkable post-operative course. A pathological examination of the right lung lobe revealed a 5 cm poorly differentiated carcinoma. Immunohistochemical stains of tumor sections revealed a high Ki-67 proliferation index, positive cytokeratin 5/6 (CK 5/6), focally positive p63 and negative p53 status.

**Figure 1:**
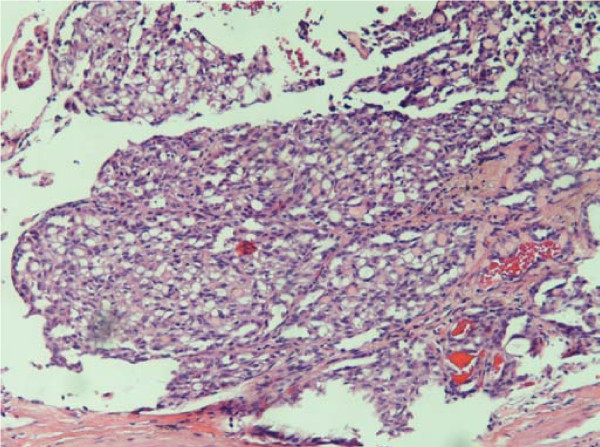
**Lesion consisting of irregular shaped spaces lined with flat cuboidal epithelium with a few spaces containing eosinophilic granular material**. The interstitium contains a myxoid matrix (hematoxylin and eosin stain; original magnification, 40 ×).

**Figure 2:**
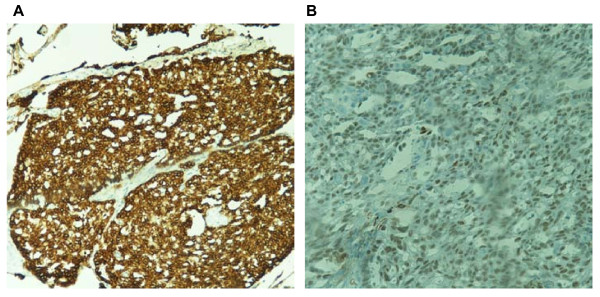
**Immunohistochemical staining showing positivity for (a) cytokeratin AE1/AE3 antibody (original magnification, 40×) and (b) thyroid transcription factor 1 (original magnification, 100×) in the epithelial cells lining the cysts**.

Incidentally, a 0.2 cm circumscribed microscopic lesion was identified on the stapled parenchymal margin of the lung lobe. Histologically, this lesion was well demarcated and composed of bland cuboidal cells and was morphologically different from the original lung carcinoma. The interstitial cellular component consisted of vaguely spindle-shaped cells (Figure 1). Considering the morphology of the lesion, a panel of differential diagnoses, including AA, adenomatoid tumor, glomangioma and tumorlet, were entertained. Immunohistochemistry revealed the lesion to stain positively for CK AE1/AE3 antibody (Figure 2a) and thyroid transcription factor 1 (TTF-1) (Figure 2b) and negatively for p53, synaptophysin, chromogranin, p63, S-100, CD31, calretenin and Wilms' tumor 1 (WT1). The combination of morphological and immunohistochemical studies confirmed the lesion to be a microscopic AA.

## Discussion

To the best of our knowledge, the coexistence of a microscopic AA with lung carcinoma has not been reported previously. Approximately 30 cases of AA, mostly incidental, have been identified in the English language medical literature to date. However, the exact number of these cases is difficult to ascertain, as these tumors are easily confused with other rare lung tumors. In 1999, Burke *et al*. [4] reported 25 true cases of AAs on the basis of histology and histochemical and immunohistochemical studies. The average size of AAs reported in the literature is 4.5 cm (ranging from 1 cm to 9 cm), with the smallest size described being 8 mm [9].

AAs are clinically important as they can imitate different specific lung diseases, focal non-specific inflammation and benign primary or secondary malignant tumors. Recognition of its characteristic morphological appearance allows for its distinction from other benign lesions of the lung. The indolent clinical progression and absence of recurrence and metastasis after complete resection are important characteristics indicative of the benign nature of AAs that are confirmed by a diploid DNA pattern visualized by flow cytometry, a low Ki-67 proliferation index and negative immunohistochemical p53 status [10]. In accordance with these findings, the benign character of the AA in our patient was evident on the basis of the immunohistochemical studies showing an absence of Ki-67 and p63 staining that was in contrast to the malignant character of the primary carcinoma of the lung diagnosed on the basis of a high Ki-67 proliferation index and a focally positive p63 status. The distinction of the behavior patterns between the two tumors on the basis of p53 status, however, was equivocal because neither of the tumors showed positivity on p53 immunohistochemical staining.

Macroscopically, AAs are well demarcated, spongy nodules located in any lobe beneath intact pleura. Not truly encapsulated, their borders are sharply demarcated from adjacent compressed lung tissue, allowing for surgical enucleation of the lesions. Most of the cases are asymptomatic [4], and a few of the patients have symptoms that are unrelated to the lesion or are pleuritic in nature. These lesions have unknown growth potential and are usually stable over a long period of time; only one case showed 20% growth during an 8-month follow-up period [11].

Small, frozen biopsy sections are usually non-diagnostic for AAs as they closely resemble normal lung parenchyma. Occasionally, they might simulate a malignancy if the small glandular spaces are lined with regular glandular epithelium [4]. CT investigation is non-specific and shows a well defined, circumscribed mass with homogeneous density. A bronchoscopy is unlikely to reveal AAs, owing to the subpleural location of these lesions. Accurate diagnosis requires correct recognition of the lesion's histological characteristics that is supported by immunohistochemical studies.

The histopathology of AAs is variable. Most AAs are well demarcated, multi-cystic and lined by a single layer of epithelial cells that are cuboidal or hobnailed in appearance [3,10]. The interstitial component usually contains collagen fibrils and spindle-shaped cells resembling fibroblasts or modified smooth muscle cells [3,4]. The cytoplasm is eosinophilic, finely vacuolated or foamy. Immunohistochemical studies have consistently shown that the epithelial cells in AAs are positive for TTF-1, and almost all of them are CK positive. Although most reports do not specify the subgroup of CKs tested, antibodies against AE1/AE3, CK1, CK7, CK18 and CK19 have been found to be positive in these tumors [3,4]. In accordance with these findings, our case demonstrated positivity for AE1/AE3 and TTF-1 and was negative for synaptophysin, chromogranin, S-100, CD31, calretenin and WT1.

The differential diagnoses of AAs are extensive, and those that need to be addressed include pulmonary adenoma, sclerosing hemangiomas, lymphangioma, atypical adenomatous hyperplasia and bronchoalveolar carcinoma [4]. A prominent papillary pattern and a heterogeneous epithelial component distinguish AAs from pulmonary adenomas. Sclerosing hemangiomas may also form cystic spaces, but they contain blood and not eosinophilic material. The TTF-1 expression observed in AAs is very important in discriminating AAs from sclerosing hemangiomas. Lymphangiomas contain lining cells that are CK negative and endothelial marker positive. Atypical adenomatous hyperplasias present an atypical bronchoalveolar proliferation, whereas AAs lack cellular atypia [4].

AAs are characterized by a proliferative alveolar epithelium and septal mesenchyma [3]. Microscopic studies [1,4] and immunohistochemical studies [5] targeted at apoproteins B and C of human surfactant demonstrate that the epithelial component is derived from type II pneumocytes. Some authors, however, have suggested that the cell of origin in AAs is probably a primitive mesenchymal cell that has the capacity to differentiate toward a type II pneumocyte [10,12]. The interstitial component varies from sparse to exuberant, showing collagen fibrils and spindle-shaped or oval cells. Immunohistochemical studies and ultrastructural studies shown in several case reports have verified that the interstitial cellular component of these tumors is made up of fibroblasts or fibroblast-like cells [4]. While proliferation of type II pneumocytes is more characteristic of AAs, mesenchymal proliferation is most likely secondary in character, being stimulated by the growth of epithelial cells, and it is seen in central zones of the tumor and as a residual lesion on its periphery.

## Conclusions

AAs usually present as an incidental solitary pulmonary mass sharing an overlap of radiographic appearance with bronchial adenomas, papillary adenomas and focal bronchoalveolar carcinomas. They are entirely benign neoplasms that can be precisely diagnosed on the basis of immunohistochemical analysis in addition to its unique histopathological characteristics. In summary, this is the first case report in the literature describing the rare presentation of AA coexisting along with lung carcinoma in the same lobe of the lung.

## Consent

Written informed consent was obtained from the patient for publication of this case report and any accompanying images. A copy of the written consent is available for review by the Editor-in-Chief of this journal.

## Competing interests

The authors declare that they have no competing interests.

## Authors' contributions

TB conceived the case report, acquired data from our patient, searched the literature and drafted the manuscript. GU performed the gross examination of the specimen and made revisions to the manuscript. JT evaluated our patient, conducted follow-up examinations, obtained our patient's consent for publication and made substantial revisions to the manuscript. CG operated on our patient, helped with the gross examination and made critical revisions to the manuscript. YH performed the histopathological evaluation of the slides and made revisions to the manuscript. JK performed the histopathological evaluation of the slides and critical analyzed the manuscript. All authors read and approved the final manuscript.
